# Blunt Liver Injury with Intact Ribs under Impacts on the Abdomen: A Biomechanical Investigation

**DOI:** 10.1371/journal.pone.0052366

**Published:** 2013-01-07

**Authors:** Yu Shao, Donghua Zou, Zhengdong Li, Lei Wan, Zhiqiang Qin, Ningguo Liu, Jianhua Zhang, Liangwei Zhong, Ping Huang, Yijiu Chen

**Affiliations:** 1 Department of Forensic Pathology, Institute of Forensic Sciences, Ministry of Justice, P. R. China, Shanghai, China; 2 Department of Forensic Science, Shanghai Medical College, Fudan University, Shanghai, China; 3 College of Mechanical Engineering, University of Shanghai for Science and Technology, Shanghai, China; Semmelweis University, Hungary

## Abstract

Abdominal trauma accounts for nearly 20% of all severe traffic injuries and can often result from intentional physical violence, from which blunt liver injury is regarded as the most common result and is associated with a high mortality rate. Liver injury may be caused by a direct impact with a certain velocity and energy on the abdomen, which may result in a lacerated liver by penetration of fractured ribs. However, liver ruptures without rib cage fractures were found in autopsies in a series of cases. All the victims sustained punches on the abdomen by fist. Many studies have been dedicated to determining the mechanism underlying hepatic injury following abdominal trauma, but most have been empirical. The actual process and biomechanism of liver injury induced by blunt impact on the abdomen, especially with intact ribs remained, are still inexhaustive. In order to investigate this, finite element methods and numerical simulation technology were used. A finite element human torso model was developed from high resolution CT data. The model consists of geometrically-detailed liver and rib cage models and simplified models of soft tissues, thoracic and abdominal organs. Then, the torso model was used in simulations in which the right hypochondrium was punched by a fist from the frontal, lateral, and rear directions, and in each direction with several impact velocities. Overall, the results showed that liver rupture was primarily caused by a direct strike of the ribs induced by blunt impact to the abdomen. Among three impact directions, a lateral impact was most likely to cause liver injury with a minimum punch speed of 5 m/s (the momentum was about 2.447 kg.m/s). Liver injuries could occur in isolation and were not accompanied by rib fractures due to different material characteristics and injury tolerance.

## Introduction

Abdominal trauma accounts for nearly 20% of all severe traffic injuries and can often result from intentional physical violence. Blunt liver injury is regarded as the most common type of injury following abdominal trauma, and is associated with a high mortality rate [1], [2]. Blunt liver injury can be caused due to a variety of mechanisms including the classical theory that the liver was lacerated by penetration of the ribs that are fractured due to blunt trauma. However, a series of cases accepted and identified by our institute revealed something different. All the victims sustained punches on the right hypochondrium by fist. They both had no history of hepatic diseases and postmortem drug and alcohol screening were negative, and most of them did not receive chest cardiac massage. During the autopsies of the victims, liver injuries with different severities were detected, but with no rib cage fractures. And some of the victims revealed no hemorrhages in both the subcutaneous tissues and thoracic and abdominal muscles of the right hypochondrium. In view of biomechanics it is not well understood why blunt liver injury occurs and why rib cage resists abdomen impact injuries in such cases.

Many studies have been dedicated to determining the mechanism underlying hepatic injury following abdominal trauma, but most have been empirical. And the hidden kinematic interactions between the liver and other abdominal organs are impossible to measure using standard biomechanical instrumentation. So the actual process and biomechanism of blunty liver injury still remain inexhaustive [1]. The recently developed finite element (FE) models and numerical simulation technology have been applied in studies of biological form and function of different animals such as gray wolf, dingo, domestic pig and woodpecker [3]–[6]. It also provides an effective tool to investigate mechanisms of injuries to the human body when combined with injury criteria and failure thresholds of different tissues.

This present study focused on blunt liver injury resulting from impact on the abdomen without rib cage fractures. FE models of the human torso have been developed to simulate blunt abdominal impact to predict liver injuries and rib cage injuries. Similar studies are compared and the biomechanism underlying blunt liver injury with intact ribs is discussed.

## Materials and Methods

The study was approved by the Science and Ethics Committee of Institute of Forensic Sciences, Ministry of Justice, P. R. China. Written informed consents were obtained from all volunteers involved in the study.

### Developing the FE model

The FE model we used in the current study was developed from high resolution CT data of the human body. The geometry of components of the whole model were reconstructed from CT data of a healthy adult male (age, 40 years; height, 175 cm; weight, 75 kg) using Mimics 14.0 (Materialise Inc., Leuven, Belgium). The same software was used to create tetrahedron meshes of the model. The volumetric mesh was imported into ANSYS Workbench 12.1 (ANSYS Inc., Canonsburg, Pennsylvania, USA.) to assign material properties, to establish boundary conditions, constrains, and loads, and to perform a preliminary solving, thus creating a keyword file. The k file was then imported into LS-PrePost 3.1 (LSTC, Inc., Livermore, CA, USA) to make modification of those aspects of the model and give analysis conditions. The analysis was performed using double precision version of LS-DYNA971 in a desk computer (Intel I7-930 CPU with 4 cores, 6G RAM).

**Figure 1:**
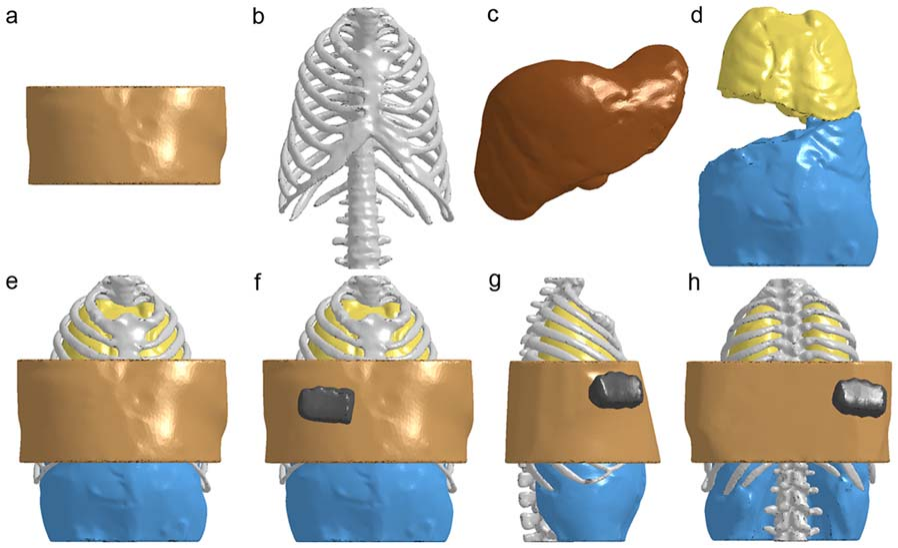
Finite Element Model of the human torso with tissue compartments and impact scenario settings. **a.** soft tissue. **b.** bone. **c.** liver. **d.** thoracic and abdominal organ combinations. **e.** the whole torso model. **f.** frontal impact scenario. **g.** lateral impact scenario. **h.** rear impact scenario.

The whole FE torso model was divided into several parts, including thoracic and abdominal soft tissues, thoracic cage, thoracic organ combination, liver, and abdominal organ combination, containing the main anatomic features (Fig. 1a–e). Because the objective of this FE analysis was to investigate the biomechanism of the liver injury, we detailed the geometry of the liver FE model and made reasonable simplification of our FE torso model. Skin, fat tissue, and muscles of the thorax and abdomen were combined into one part. The pleural, lungs, mediastinum, heart, and diaphragm were also made into a whole part. The abdominal part was combined from the peritoneum, spleen, and other abdominal organs. The ribs were separated into cortical and trabecular bones, and all of the bony structures of the sternum and vertebrae were modeled as one part for simplicity. The soft tissue part was reduced in height to cover a certain area outside of the liver for the same reason. We also developed a FE model of a fist from CT data. The average element length was 10 mm. Other meshing parameters, including warpage, aspect ratio, skew, and Jacobian, were set based on the literature [7]. The whole torso model consisted of 1701986 solid elements and 381376 nodes, and the liver model consisted of 366295 elements and 63045 nodes. The mean element quality was >0.7.

The material properties assignment is detailed in Table 1. Ribs were assigned an elastic-plastic material model [8] and a hyperelastic model [12] was used to simulate liver behaviors under impact. The fist was defined as a rigid body and the effective mass was 489.393 g. We also made several simplifications in characterizing material properties of tissues in order to keep anatomic and biomechanical features of the tissues, while saving computation time.

**Table 1:** Material properties used in the FE model.

Parts	Material type	Parameters	Reference
Rib (cortical)	Elastic-plastic	ρ = 2.0 g/cm^3^; E = 11.5 GPa; μ = 0.3; σ_y_ = 88 MPa; E_t_ = 1.15 GPa; β = 0.1; C = 2.5, P = 7 (Cowper-Symonds model); Plastic failure strain = 0.02	Li et al. [8]
Rib (trabecular)	Elastic-plastic	ρ = 1.0 g/cm^3^; E = 0.04 GPa; μ = 0.45; σ_y_ = 2.2 MPa; E_t_ = 0.001 GPa; β = 0.1; C = 2.5, P = 7 (Cowper-Symonds model); Plastic failure strain = 0.03	Li et al. [8]
Sternum	Elastic-plastic	ρ = 2.0 g/cm^3^; E = 9860 MPa; μ = 0.3; σ_y_ = 66.7 MPa;	Shigeta et al. [7]
Vertebrae	Elastic-plastic	ρ = 2.0 g/cm^3^; E = 12000 MPa;μ = 0.3; σ_y_ = 100 MPa;	Shigeta et al. [7]
Muscle	Elastic	ρ = 0.9 g/cm^3^; E = 0.5 MPa;	Shigeta et al. [7]
		μ = 0.43;	Mollemans et al. [9]
Liver	Hyperelastic	ρ = 1.04 g/cm^3^;	Gao et al. [10]
		μ = 0.49;	Leroy et al. [11]
		C10 = C01 = 6206 Pa; C20 = C02 = 3492 Pa; C11 = 0;	Miller [12]
Thoracic organ combination	Elastic	ρ = 0.2 g/cm^3^; E = 5 kPa;	Cronin [13]
		μ = 0.45;	Brock et al. [14]
Abdominal organ combination	Elastic	ρ = 1.0 g/cm^3^; E = 13 MPa;μ = 0.4;	Shigeta et al. [7]

### Other modeling considerations

With respect to the boundary conditions, sliding and frictionless contacts were implemented between the surfaces of the liver and the thoracic and abdominal organs inside the body cavities, allowing for relative movement and force transmission of organs during the impact. The same contact model was used between the soft tissue and visceral parts. In addition, a sliding and friction contact was modeled between the soft tissue and bony structures, including the thoracic cage and fist. Frontal, lateral, and anterior-posterior loading tests based on the literature [8], [15], [16] were used to validate the rib cage part of the FE torso model and a large deformation test by Nava et al. [17] was used to validate the FE liver model.

### Validation of the liver model

**Figure 2:**
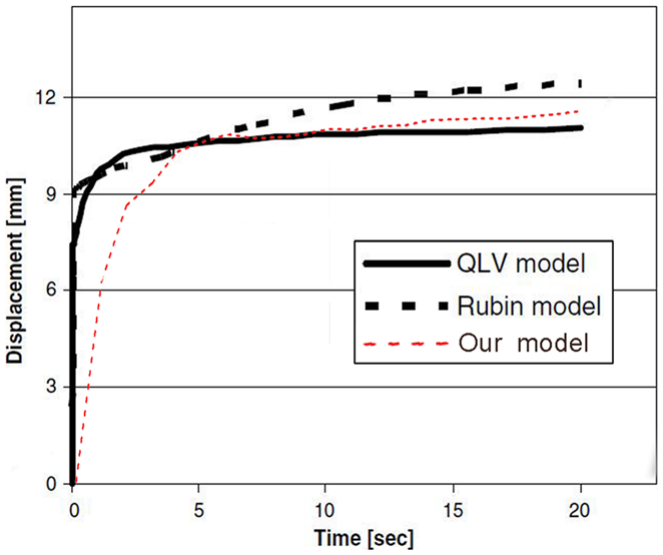
Time history of the maximum displacement of liver with a concentrated load of 2 N applied.

Our FE liver model was validated against the experiment of Nava et al. [17]. According to their experimental procedure, the upper part of the right lobe of the liver model was fully constrained and a constant concentrated force of 2 N was applied locally on the surface of the quadrate lobe. The simulation time was set to 20 s, and the displacement time history over the 20 s was output (Fig. 2). It can be seen from the displacement time history that the displacement-time curve of our liver model showed good agreement with the curves obtained in Nava's experiments. Our FE liver model has been validated to be available in the FE simulations.

### Impact scenario settings

The torso model was set to be punched in the right hypochondrium by the fist from the frontal, lateral and rear directions (Fig. 1f–h), and in each direction with impact velocities of 4, 5, 6, 7, and 8 m/s. During the impact process, only the lower part of the spine was fixed. The termination time was set to 10 ms and the time interval between outputs was set to 0.1 ms.

## Results

The computation time lasted approximately 80 hours for each impact simulation. The deformation, displacement, reaction force, stress, and strain distribution of the model parts were calculated and used to analyze the biomechanism underlying hepatic injury. The results are summarized in Table 2.

**Table 2:** Summary of simulation results.

	Velocity (m/s)	Momentum (kg.m/s)	Liver Injury Time (ms)	Maximum Principal Strain of Liver^a^	Peak Maximum Principal Strain of Liver^b^	Contact Force (N)^b^^,^ c	Contact Force (N)^b^^,^ d
Frontal Impact	4	1.958	No Injury	No injury	0.250	534	5.7
	5	2.447	No Injury	No injury	0.280	700	9.3
	6	2.936	3.3	0.362	0.513	852	12.0
	7	3.426	3.1	0.319	0.582	1000	15.8
	8	3.915	2.7	0.393	0.696	1138	17.9
Lateral Impact	4	1.958	No Injury	No Injury	0.277	378	3.2
	5	2.447	3.8	0.131	0.333	506	5.9
	6	2.936	3.3	0.329	0.546	636	9.9
	7	3.426	3.0	0.327	0.626	769	15.8
	8	3.915	2.7	0.346	0.701	898	22.9
Rear Impact	4	1.958	No Injury	No Injury	0.020	426	<0.1
	5	2.447	No Injury	No Injury	0.058	550	0.1
	6	2.936	No Injury	No Injury	0.093	697	0.2
	7	3.426	No Injury	No Injury	0.108	840	0.5
	8	3.915	No Injury	No Injury	0.126	976	0.7

a Results were calculated when liver injury occurred.

b Results were calculated after the whole impact process. Contact forces between the entire body parts with multiple contact areas were predicted.

c Contact forces between fist and muscle.

d Contact forces between ribs and liver.

### Frontal Impacts with Different Velocities

**Figure 3:**
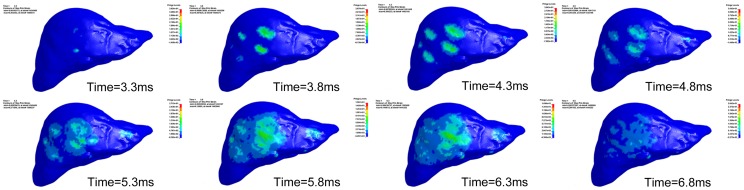
The time course of the maximum principle strain distribution in the liver. The abdomen was under frontal impact with a velocity of 6 m/s.

In each impact velocity scenario, the overall kinematics of the torso showed that the rib cage and the surrounding soft tissue were pressed and compressed inside in the first several milliseconds after the impact, then rebounded with the fist. The liver was impacted and compressed by the anterior chest wall beneath the impact area of the body surface. Strain in the liver was concentrated in the anterior side of the right lobe corresponding with the contact positions of the ribs. At the impact velocities of 4 and 5 m/s, the predicted peak maximum principle strain of the liver reached 0.28, which did not exceed the liver injury threshold of 0.3 [7], thus no injury was predicted. The maximum principle strain first reached 0.362 in approximately 3.3 ms after the initial impact with a velocity of 6 m/s (the momentum was about 2.936 kg.m/s). The liver was predicted to be injured in the area in contact with the ribs. During the entire process, several areas were predicted to be injured in succession. The time course of the maximum principle strain distribution in the liver is illustrated in Fig. 3. At velocities of 7 and 8 m/s, the liver injury was first simulated to occur at 3.1 and 2.7 ms after the initial impact, respectively, which was nearly in the same area as the 6 m/s scenario. No rib fractures were observed under all impact velocities because the failure strain of the ribs never reached the injury threshold of 0.02 [8]. In additional, the impact force to the muscle was measured as 700 N at a punch speed of 5 m/s, while the impact force transmitted to the liver through the rib cage was measured as 9.3N.

### Lateral Impacts with Different Velocities

**Figure 4:**
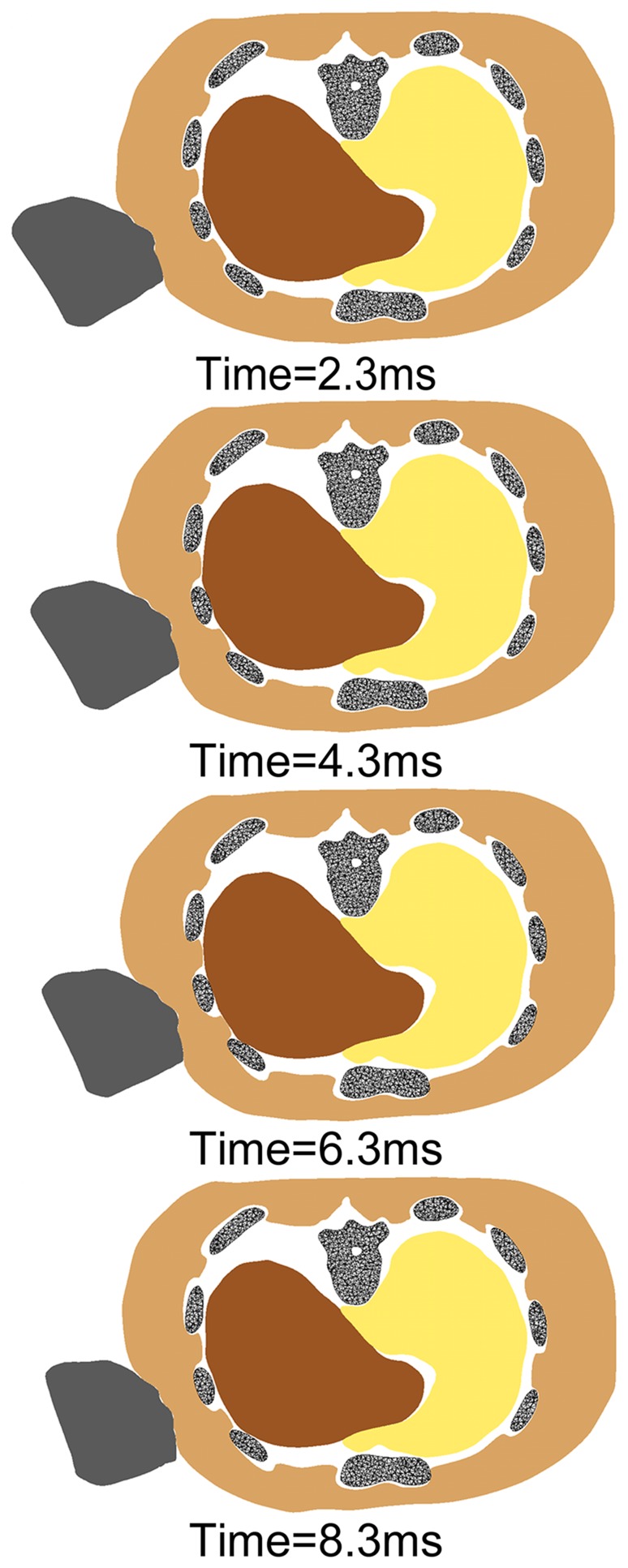
The overall kinematics of the FE human torso model during the lateral impact scenario. The rib cage and soft tissues were compressed and then rebounded. The liver was impacted by the ribs and was locally compressed.

Similar to the kinematics of the frontal impact scenario, the rib cage and soft tissues were compressed first, and then rebounded during the 10 ms process, but with a larger range of motion. The liver was impacted by the ribs several milliseconds after the punch and was locally compressed. No liver injury was predicted at an impact velocity of 4 m/s. With a 5 m/s punch (the momentum was about 2.447 kg.m/s), hepatic injury was first predicted in the side of the right lobe where the liver was directly hit by the lateral chest wall. Liver injury occurred at 3.8 ms after the impact when the maximum principle strain reached 0.312. The overall kinematics during the 10 ms is illustrated in Fig. 4. The time when the liver was predicted to be injured was earlier as the impact velocity increased, with the injury area nearly the same. At a 5 m/s impact velocity, the impact forces to the muscle and liver were predicted to be 506 N and 5.9 N, respectively. No rib fractures were predicted under all impact velocities.

### Rear Impacts with Different Velocities

With a rear impact, the rib cage and soft tissues had a significantly smaller range of motion than in the other two directions. Unlike the situations in the other two directions, slight contact was observed between the liver and the posterior chest wall, but local contact between the liver and spine was noticed, as reflected by a certain strain concentration in the posterior side of the liver corresponding with the contact area of the vertebrae. No rib fractures or liver injuries were predicted in all velocities with a rear impact. Even under an impact velocity of 8 m/s, the peak maximum principle strain of the liver just reached 0.126, but never exceeded the threshold value.

### Liver Injury Mechanisms by Comparing Impacts in Different Directions

The minimum impact velocity under which the liver injuries may occur were predicted as 6 m/s, 5 m/s, and >8 m/s in the frontal, lateral, and rear directions, respectively. According to the kinematics of the whole torso FE model and the strain distribution in the liver, a likely mechanism of liver injury in all impact direction scenarios could be impact loading from the rib cage, which resulted from a punch to the body surface. A liver injury most likely occurred in the lateral impact with a velocity of 5 m/s (2.936 kg.m/s in momentum). Under the same impact direction, the higher the initial velocity and momentum, the earlier the injury occurs, resulting in higher maximum principle strain values in the injury area. In all simulations, ribs showed compression and rebound during the impact process but no rib fractures were predicted.

## Discussion

Identifying the mechanisms of injury under different injury situations is an essential for forensic pathologists, not only for cranial-cerebral injuries, but also for blunt force abdominal injuries, such as a ruptured liver. Traditional research has focused on hepatic injuries, as hepatic injuries are associated with a high injury frequency and mortality rate among patients with abdominal trauma [1].

Most of the investigations involving mechanisms of liver injury have been based on experiments involving animals, human bodies, or cadavers relating abstract quantities, such as impact force, velocities, and abdominal compression to injuries observed at the time of autopsy, which are mostly empirical and contribute little to the actual process and biomechanism of hepatic injury. Due to the consideration of inherent variability of human anatomy and soft tissue properties, FE models and numerical simulation technology have been shown to be effective and powerful tools to investigate mechanisms of organ injury and human tolerance to a wide range of traumatic impact.

Several studies have been conducted to investigate the biomechanism of blunt injury of organs using FE models of humans. Richens et al. [18] and Grimal et al. [19] have performed FE studies to investigate the biomechanism of blunt traumatic aortic rupture and lung injury under high-speed blunt impact, respectively. Snedeker et al. [1], [20] performed a series of studies to explore comprehensive renal injury concepts under blunt trauma using FE models of the human kidney and abdomen; however, research articles involving the development of a FE human liver model and biomechanical analysis of blunt hepatic injury are limited. Shigeta et al. [7] developed a human body FE model and the model was validated to be capable of internal organ injury prediction in car crashes. Thoracic and abdominal impactor tests were simulated in the validation process, and injuries in different organs were predicted under those simulations, including hepatic injuries. Shigeta et al. [7] focused on the mechanical response of the entire torso model against anterior loading, but the actual process and biomechanism of hepatic injury under blunt impact from different directions, velocities, momentum and hit locations were not discussed. Tungjitkusolmun et al. [21] and Nava et al. [17] conducted their researches using three-dimensional FE analysis; all the researches were for surgical simulation purposes or in constructing constitutive models for the liver, but not for identifying the biomechanism underlying hepatic injury with respect to forensic pathology.

For the first time, we have described a definite injury concept of the liver under blunt impact based on FE analysis, specifically with respect to punches to the abdomen. Development of a three-dimensional human torso FE model, which consists of geometrically-detailed liver and rib cage FE models and a simplified representation of other thoracoabdominal organs, was used to simulate fist punches to the right hypochondrium with five different impact velocities and three impact directions. The biomechanical response of the model, including the dynamic process of deformation and compression, strain distribution and concentration, and contact force were taken into account to analyze the biomechanism underlying liver injury, and development of a concept for blunt liver injury.

The mechanisms of blunt liver injury range from simple compression against the spine or posterior wall of the abdomen in low-velocity impacts, to viscous injury caused by accumulation of internal fluid pressure at high rates of loading, leading to excessive tensile or shear strains. The liver may also be lacerated by penetration of the ribs that are fractured due to blunt trauma [22].

According to the overall kinematics and time course of strain distribution of the liver in 10 ms increments, the liver had local compression in parallel with strain concentration at the contact area after being struck by the ribs, then energy was spread to the surrounding tissues in the form of waves. The shapes of high-strain areas in the liver match the geometry of the ribs during the simulated impact process. We consider the direct impact by the ribs to be the most likely mechanism underlying liver injury following blunt insults, such as a punch to the abdomen. The impact force and energy were translated to the liver through muscles and the rib cage in succession, leading to strain concentrations of high values in the liver, and injury occurs at the time that strain values exceeded the injury threshold, as was elaborated in the research by Grimal et al. [19].

The assumption of using maximum principal strain as the injury criterion for liver in our research was referred to the research of Shigeta et al. [7]. And they obtained such an injury criterion from a study by Melvin et al. [23] in 1973. In the animal experiments conducted by Melvin et al., the liver of the Rhesus monkey was surgically mobilized and was laid onto a small load cell. A platen with controlled velocity and penetration depth was impacted downwards to apply a compression load to the liver. The strain was obtained by dividing the indentation depth by the initial height of the liver where being contacted with the platen. In fact the strain obtained was a nominal strain. According to the definition of principal strain, the liver actually had principal strains in all the three directions when uniaxially compressed. Due to the very small contact area between the platen and the liver, the other two principal strain values other than the maximum principal strain value were very small. Theoretically, when the values of the other two principal strains were very small, the nominal strain value was approximately the same as the maximum principal strain value. So we believe that the compression strain obtained in Melvin's experiment was a nominal strain and it was also certain that the maximum principal strain value approximated the nominal strain value, for the other two principal strain values had little impact on the calculation. Also, judged from the time course of strain distribution in liver which was illustrated in the article of Shigeta et al., the strain they used for criterion was refer to a local strain which was probably the maximum principal strain. So we consider that the assumption of using maximum principal strain as an injury criterion for liver was reasonable, credible and practicable in our research.

In addition, the simulated results also revealed compression and strain concentration on the posterior side of the liver, when the liver had moderate contact with the posterior chest wall and spine during the 10 ms process. The anterior chest wall was compressed inward during the punch, and because of the special anatomic relationships of the liver, the mobility was confined to the area between the posterior chest wall and spine. In addition to the direct impact loading by the ribs, compressive loading between the anterior and posterior rib cage and the bony surface of the spine may also contribute to the liver injury.

According to the cases, no lesion was detected in the bones during the autopsy on the victims, with the exception of an apparent liver rupture below the punch area to the body surface, which was considered lethal. And in some cases the victims' skin, subcutaneous tissues and muscles were also clean. In such cases the hepatic injuries were due to the transmission of impact force and energy from the thoracic wall surface to the liver and the specific mechanisms were elucidated by the FE simulations. Simulation results indicated that among punches from three different directions to the right hypochondrium, lateral impact loading was most likely to cause liver injury with a minimum punch speed of 5 m/s (the momentum was about 2.447 kg.m/s). The injury mechanism refers to the ever-increasing maximum principle strain, which is a possible injury criterion, in the liver exceeds the injury threshold, driven by the motion of the thoracic wall. We suggest that the mechanism of injury can be attributed to the geometric characteristics of the rib cage. As distinct from the anterior and posterior parts of the rib cage which have fixed joints with the sternum and vertebrae, the lateral parts of the ribs possess a higher mobility and usually undergo a wider range of motion when sustaining the same impact energy.

**Figure 5:**
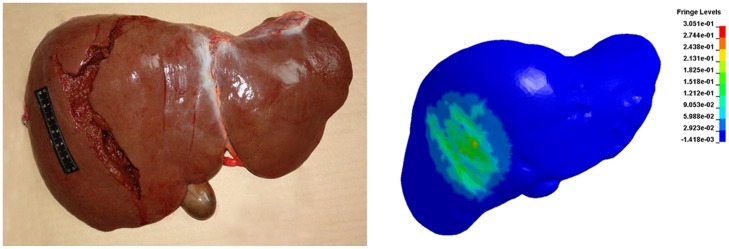
The comparison between the liver rupture of the victim and the simulated results. Results of lateral punch simulations with an impact velocity of 6 m/s were illustrated. High gradients of colour indicate region of greatest strain and location of injuries.

According to the simulated results, local hepatic injury was predicted at a 5 m/s lateral punch simulation, but both range and severity were too mild to be considered lethal. Lateral punch simulations with impact velocities >6 m/s (momentum>2.936 kg.m/s) resulted in wide ranges of hepatic injuries in the right lobe, which is consistent with the liver rupture of the victim in one of our cases (Fig. 5).

For the given lateral punch simulations, the contact force between the fist and thoracoabdominal muscles was calculated as 636 N at a punch velocity of 6 m/s, but being merely 9.9 N between the rib cage and liver under the same conditions. The vast amount of the initial impact energy was absorbed and dissipated in the soft tissues and bony structures, leaving only a minor part transmitted to the liver. Although the value of impact force transmitted to the liver decreased to approximately 1.6% of the initial impact force sustained by the muscles and rib cage, the pressure on the injury area of the liver was predicted as 44 kPa when injury occurred. It was reported in the literature that a peak tissue pressure of 48 kPa was correlated to 50% risk of serious (AIS> = 3) liver injury [24], so we believe that when applied to a small area, such little force is also capable of causing liver injury due to the hyperelastic material properties. At the same time, the muscles and rib cage remained intact due to the material characteristics, which could sustain larger amounts of impact energy. The values of injury criteria of those tissues are maintained below the threshold level during the entire punch process, thus no lesion was predicted.

The general theory of liver injury mechanism also suggests that the liver may be injured by motion relative to the rest of the body during rapid deceleration [22]. Snedeker et al. [1] supported the theory through the impact simulation that renal injury may result during secondary interactions of the blunty impacted kidneys in the abdominal cavity. In such cases, organ injury is typically at points of attachment and is caused by stretching the ligaments or blood vessels beyond the tensile strength. Due to the simplification of the model that sliding and frictionless contacts of the liver without attachment to the combined thoracic or abdominal structures were made, the model could not effectively predict such decelerated motion or secondary interactions of the liver. However, the autopsy findings of the victims did not reveal typical liver injuries relative to adjacent tissues. Rapid deceleration of the liver is more common in cases like falls or traffic accidents, and under such circumstances the abdomen usually strikes against a large flat surface. In our cases, liver rupture was involved in isolated blunt force impact to the abdomen with a small contact area, therefore the mechanism of injury should simply be the direct impact by the accelerated ribs; compressive loading by the rib cage and spine may also be contributory. The probability of extrathoracic heart massage-induced injury was also excluded.

It should be noted that several simplifications were made in developing the FE torso model which may affect the biomechanical behavior under blunt impact. The muscle part of the model was incrassated because it was combined with skin and subcutaneous fat, thus improving the ability to buffer the impact energy. Also, the intercostal muscles were not separated from the thoracoabdominal muscles, although difference existed in material characteristics. But according to the simulated results, the predicted failure strain of the ribs was far from the injury threshold. The membrane surrounding the liver and the attached ligaments could also to some extent protect the liver from injury during blunt impact, which was not detailed in the torso model. We believe the slight simplification on constitution and mechanical properties of the model do not change in considerable proportions of the biomechanical response of the model. And the model is capable of predicting the liver injury resulting from direct loading through the rib cage. Positive modifications of the model including separating the thoracic and abdominal soft tissues and organs will be made in our future studies.

In sum, we developed a three-dimensional FE human torso model from CT data, which included detailed FE liver and rib cage models. The FE model was used to investigate the biomechanism underlying blunt liver injury through simulation of punching the abdomen and the results were analyzed in aspects of deformation, displacement, reaction force, stress, and strain distribution. The mechanism of liver injury under blunt impact to the abdomen was concluded to be primarily due to a direct strike by the ribs. The severity of injury differed with the punch direction, and a lateral impact was most likely to cause liver injury with a minimum punch speed of 5 m/s (the momentum was about 2.447 kg.m/s). The liver injury was not necessarily accompanied by rib fractures due to the material characteristics and injury tolerance. The FE models and numerical simulations are a powerful and effective method to explore the mechanism underlying injury.
